# Deciphering functional groups of rumen microbiome and their underlying potentially causal relationships in shaping host traits

**DOI:** 10.1002/imt2.225

**Published:** 2024-07-15

**Authors:** Ming‐Yuan Xue, Yun‐Yi Xie, Xin‐Wei Zang, Yi‐Fan Zhong, Xiao‐Jiao Ma, Hui‐Zeng Sun, Jian‐Xin Liu

**Affiliations:** ^1^ Institute of Dairy Science, College of Animal Sciences Zhejiang University Hangzhou China; ^2^ Xianghu Laboratory Hangzhou China; ^3^ Ministry of Education Key Laboratory of Molecular Animal Nutrition Zhejiang University Hangzhou China

**Keywords:** carbohydrate metabolism, functional groups, holo‐omics, rumen microbiome, *selenomonas bovis*

## Abstract

Over the years, microbiome research has achieved tremendous advancements driven by culture‐independent meta‐omics approaches. Despite extensive research, our understanding of the functional roles and causal effects of the microbiome on phenotypes remains limited. In this study, we focused on the rumen metaproteome, combining it with metatranscriptome and metabolome data to accurately identify the active functional distributions of rumen microorganisms and specific functional groups that influence feed efficiency. By integrating host genetics data, we established the potentially causal relationships between microbes‐proteins/metabolites‐phenotype, and identified specific patterns in which functional groups of rumen microorganisms influence host feed efficiency. We found a causal link between *Selenomonas bovis* and rumen carbohydrate metabolism, potentially mediated by bacterial chemotaxis and a two‐component regulatory system, impacting feed utilization efficiency of dairy cows. Our study on the nutrient utilization functional groups in the rumen of high‐feed‐efficiency dairy cows, along with the identification of key microbiota functional proteins and their potentially causal relationships, will help move from correlation to causation in rumen microbiome research. This will ultimately enable precise regulation of the rumen microbiota for optimized ruminant production.

## Highlights

- We combined rumen metaproteome, metatranscriptome, and metabolome data to identify active functional distributions of rumen microorganisms influencing feed efficiency.
- By integrating host genetics data, we identified causal relationships between microbes, proteins/metabolites, and phenotypes.
- We found a causal link between *Selenomonas bovis* and rumen carbohydrate metabolism, suggesting its role in improving feed utilization efficiency in dairy cows.

## Graphical abstract

In this study, we focused on the rumen metaproteome, combining it with metatranscriptome and metabolome data to identify the active functional distributions of rumen microorganisms and specific functional groups that influence feed efficiency. By integrating host genetics data, we established potentially causal relationships between microbes, proteins/metabolites, and phenotypes. We identified specific patterns in which functional groups of rumen microorganisms influence host feed efficiency. We found a causal link between *Selenomonas bovis* and rumen carbohydrate metabolism, potentially mediated by bacterial chemotaxis and a two‐component regulatory system, impacting feed utilization efficiency in dairy cows. Our study on nutrient utilization functional groups in the rumen of high‐feed‐efficiency dairy cows, along with identifying key microbiota functional proteins and their potential causal relationships, will help move from correlation to causation in rumen microbiome research. This will enable precise regulation of the rumen microbiota for optimized ruminant production.

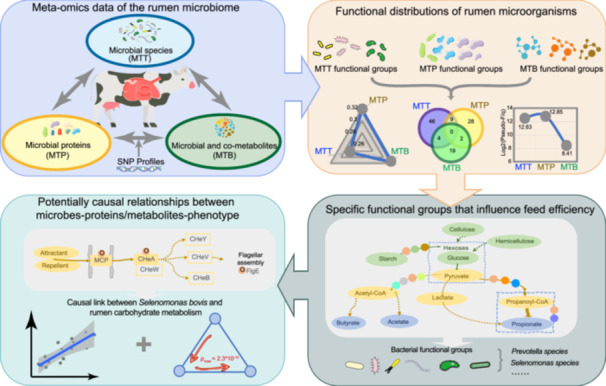

## INTRODUCTION

The rumen, the key digestive organ in ruminants like dairy cows, relies on microorganisms for feed digestion [1], significantly affecting nutrient supply and host traits [2, 3]. The rumen microbiome is responsible for degrading human‐inedible plant biomass to produce high‐quality protein products from meat or milk, while generating considerable methane that impacts the environment. Due to its complex taxonomy, large functional redundancy (FR), and strict anaerobic nature, the current understanding of the rumen microbiome is still limited. Rumen microorganisms primarily contribute to the breakdown, utilization and metabolic efficiency of dietary substrates, influencing the supply of milk precursors and energy for dairy cows, thereby impacting their milk production efficiency [3, 4, 5]. Therefore, understanding of the digestion and utilization of nutrients in the rumen is the key to targeted manipulation of rumen microorganisms and improving feed conversion efficiency in dairy cows.

Many exploratory efforts have been made toward manipulation of rumen microorganisms, deepening our understanding of the interactions between rumen microorganisms and the efficiency of feed utilization [6, 7]. Early studies on rumen metagenomics focused more on the differences in the species classification of microorganisms [6, 7]. Decoding rumen microorganisms' functions at metagenome and metatranscriptome (MTT) levels limits researchers to potential DNA‐level functions (including dead microbes) and transcription‐level functions (hindered by mRNA enrichment and RNA instability), preventing accurate functional descriptions [8]. With the advent of metaproteomics, it has become possible to describe the real functions of the rumen microbiome rather than its potential [9]. Despite some initial progresses have addressed the evaluation of protocols for metaproteomic studies focusing on the rumen microbiota [10, 11], rumen metaproteomics (especially of dairy cattle) is still in the nascent stage.

Recent advances in high‐throughput sequencing have significantly progressed the study of the rumen microbiome and its interactions with host animals [12, 13, 14]. However, the high taxonomic diversity in microbial communities raises questions about species coexistence and its impact on community function [15]. FR is a common phenomenon among taxonomically distinct microorganisms that encode the same energy‐yielding metabolic functions and FR contradicts the expectation that each species should occupy a distinct metabolic niche [15]. The taxa encoding each function can vary across space and time with minimal impact on the function itself, often due to ecological drift among equivalent organisms. The rumen microbiome's complex communities and feed efficiency remain largely unexplored. Much remains to be discovered about FR and the functional groups within the rumen microbiota. Therefore, our current study focused on the rumen metaproteome (MTP), using MTT data as the database to accurately characterize the true functional distribution of rumen microorganisms and to reveal their proteins that play different roles. Integrative “holo‐omics” approaches that combine metadata with DNA, RNA, and protein data from both host and microbes are expected to uncover hidden functional interactions. We included three levels of meta‐omics data (MTT, MTP, and metabolome [MTB]) together with host genetics data and identified microbial functional groups that were closely related to feed efficiency, with their causal relationships underlined. The outcomes from this study will provide a new perspective for the functional characterization of rumen microorganisms.

## RESULTS

### Overview of meta‐omics data of the rumen microbiome

**Figure 1:**
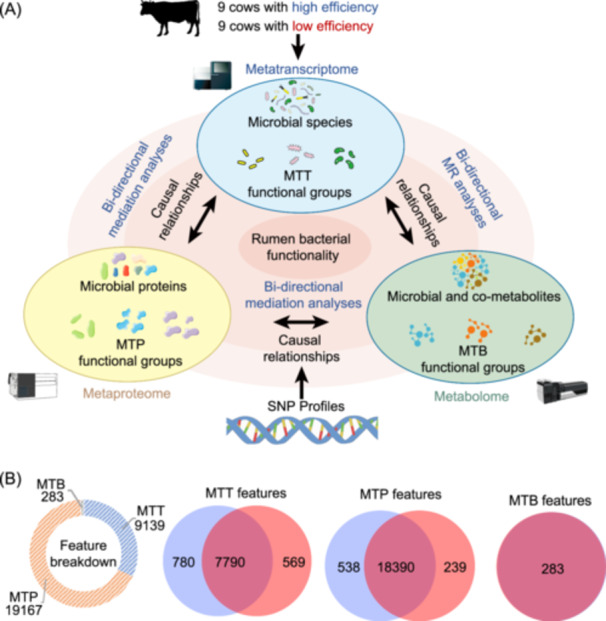
A systematic workflow and feature overview. (A) Pipeline overview of the ruminal metatranscriptomics (MTT), metaproteomics (MTP), and metabolomics (MTB) of experimental dairy cows. (B) Venn diagram showing meta‐omics feature overlaps of the efficient and inefficient cows. We performed the rumen metaproteome, combining it with metatranscriptome and metabolome data to accurately identify the active functional distributions of rumen microorganisms and specific functional groups that influence feed efficiency. By integrating host genetics data, we established the causal relationships between microbes‐proteins/metabolites‐phenotype, and identified specific patterns in which functional groups of rumen microorganisms influence host feed efficiency. Blue: efficient animals, red: inefficient animals. SNP, single nucleotide polymorphisms.

To analyze the functionality of the rumen microbiota at different levels, our study included MTT, MTP, and MTB data from rumen samples from 9 high‐ and 9 low‐feed efficiency dairy cows (Figure 1 and Table S1). A total of 28,589 features from three different omics platforms were initially identified, and we included a total of 20,571 single nucleotide polymorphisms (SNP) loci from 298 dairy cattle as a mediating factor for investigating the potentially causal relationship behind the omics‐based relationships (Figure 1A). MTT sequencing revealed a total of 9139 microbial taxa, and we included 283 metabolite features from untargeted mass spectrometry in the MTB data. With the MTT data used as our sample‐specific database for database searching, our MTP feature set included a total of 19,167 proteins. Given our interest in feed efficiency differences, we generated Venn diagrams distinguishing omics data between the two groups (Figure 1B). The high‐efficiency animals possessed 780 unique MTT features, while the low‐efficiency animals had 569 unique MTT features. Among the MTP features, 538 were unique to the high‐efficiency animals, and 239 were exclusive to the low‐efficiency animals. All the MTB features were shared by both the high‐ and low‐efficiency animals.

For metaproteomics, a total of 1,663,161 spectra were generated from mass spectrometry, with 475,130 spectra confidently matched to peptide sequences in the database, resulting in 85,693 identified peptides. Of these peptides, 50,096 were unique to a single protein in the database. Based on the identified peptides, we inferred 19,167 proteins in the microbial community. Among these proteins, 9848 were quantifiable proteins that could be accurately quantified based on the abundance of their corresponding peptides. After database searching, the data generated from mass spectrometry underwent a series of quality control steps, including the analysis of peptide length distribution, peptide count distribution, molecular weight distribution, and coverage distribution, to ensure that the quality of the results met standards. Most of the peptides identified by mass spectrometry had an amino acid length of 7‐20, consistent with the expected pattern for trypsin digestion and higher‐energy collision dissociation (HCD) fragmentation. Most of the proteins were identified by at least two peptides, with coverage typically below 20%.

### Functionality of rumen microbiota based on metaproteomics

**Figure 2:**
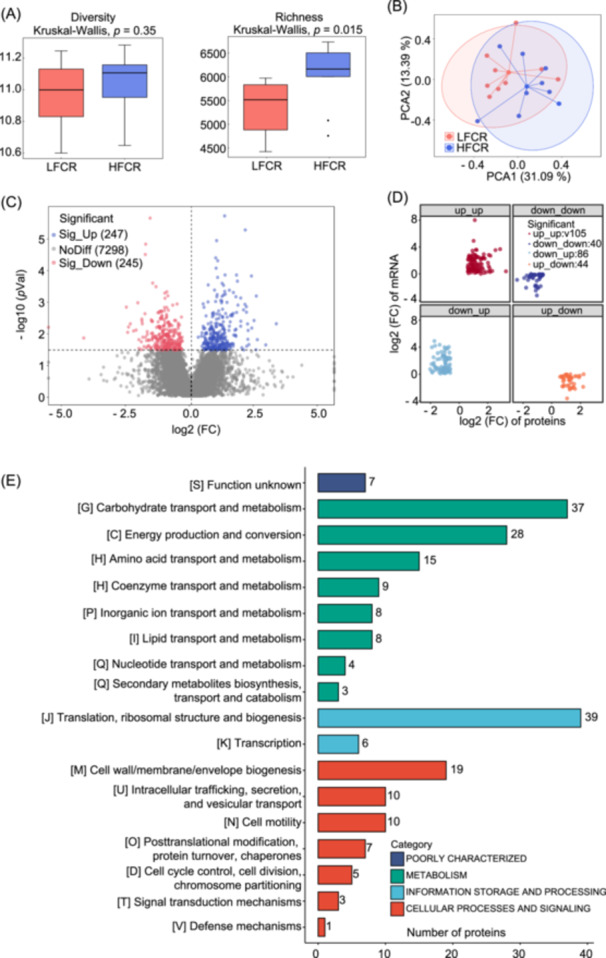
Main metaproteome features of the rumen microbiota. (A) Box plot representing alpha diversity and richness indices of the metaproteome from the rumen of cows with differential feed efficiencies. (B) Principal component analysis (PCA) plot representing beta diversity of metaproteomic data from rumen microbes of cows with differential feed efficiencies. (C) Volcano plot comparing proteins from rumen microbes of cows with differential feed efficiencies. (D) Correlation analysis between significantly differential transcriptomic features and significantly differential proteomic features. (E) Functional classification of significantly upregulated cluster of orthologous groups of protein (COGs). FC, fold change; HFCR, high feed conversion ratio; LFCR, low feed conversion ratio.

With the advent of metaproteomics, it has become possible to describe the real functions of the rumen microbiome rather than its potential. Our analysis of the functional diversity of the rumen microbiota at the metaproteomics level showed no significant difference in the Shannon index between the high‐ and low‐efficiency groups (*p* > 0.05), but the Chao1 richness index was significantly higher in the high‐efficiency group (*p* = 0.015) (Figure 2A). The principal component analysis (PCA) scatter plot showed a trend of separation between the high‐ and low‐efficiency groups (Figure 2B), and the Bray‒Curtis‐based β‐diversity analysis revealed significant differences in the microbial community structure between the two groups (corrected by permutational multivariate analysis of variance, permutations = 999, *R* ^2^ = 0.32, *q* < 0.05). We identified 247 upregulated and 245 downregulated differentially expressed proteins (DEPs) in the high‐efficiency group compared with the low‐efficiency group (feed conversion ratio [FCR], adjusted *p* < 0.05, Figure 2C). Spearman correlation analysis did not reveal any significant correlation between differentially expressed genes (DEGs) and DEPs in the rumens of high‐ and low‐efficiency animals (*p* > 0.05, Figure 2D). When the results of the cluster of orthologous groups (COG) functional annotation of DEPs were summarized as a bar graph, we found that the majority of the differential proteins were involved in metabolism and cellular processes and signaling, followed by information storage and processing (Figure 2E). Overall, our findings provide important insights into the functional differences in the rumen microbiota between high‐ and low‐efficiency cows and may facilitate the development of effective strategies for optimizing rumen microbial function.

### The difference between potential and actual functional groups of rumen microbiota

**Figure 3:**
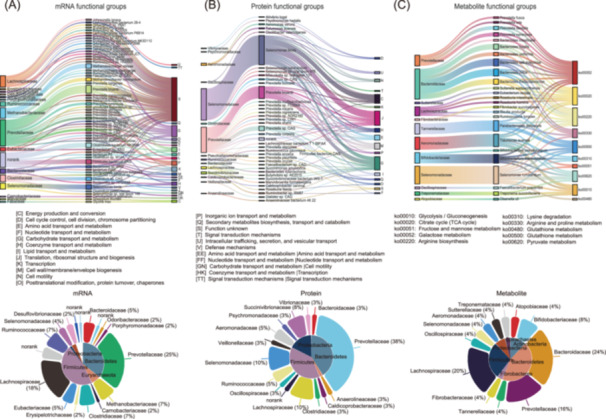
Comparison of rumen microbial functional groups related to high feed efficiency in dairy cows based on metatranscriptomics and metaproteomics. (A) Sankey diagrams of the taxonomic and functional mapping relationships of rumen microbial functional groups related to high feed efficiency, analyzed based on metatranscriptomics data. (B) Sankey diagrams display taxonomic and functional mapping relationships analyzed based on metaproteomics data. (C) Taxonomic and functional mapping relationships analyzed based on metabolomics. The stacked pie charts show the distribution of microbial functional groups based on metatranscriptomics and metaproteomics. TCA, tricarboxylic acid cycle.

To investigate the functional correlation of DEPs within each comparison group, we conducted clustering analysis and categorized the DEPs into four quartiles (Q1 to Q4) based on their fold change (Figure S1A). We then performed Gene Ontology (GO) classification, Kyoto Encyclopedia of Genes and Genomes (KEGG) pathway analysis, and protein domain enrichment analysis for each quartile and revealed the functional correlation of proteins with varying degrees of differential expression (Figure S1). Functional enrichment analyses were performed on mRNA‐based and protein‐based functional groups (MTT and MTP) using Q3 and Q4, which were significantly enriched in high feed efficiency individuals. Taxonomic information for the rumen microbial species was traced back, leading to the identification of functional groups of rumen microbes associated with high feed efficiency in dairy cows. The Sankey diagram illustrates the functional‐microbe‐taxonomy classification mapping for potential functional groups based on mRNA (Figure 3A) and actual functional groups based on protein (Figure 3B). The results of the Sankey diagram mapping, regardless of whether it was based on potential or actual functional groups, showed high redundancy at the functional level of rumen microbial communities.

The MTT‐based functional groups consisted of 59 microbial species from 17 families, with microbes from *Prevotellaceae* (25%), *Lachnospiraceae* (18%), *Ruminococcaceae* (7%), and *Clostridiaceae* (7%) being the most abundant (Figure 3A and Table S2). *Prevotella bryantii*, *Selenomonas bovis*, *Clostridium* spp., and *Firmicutes* spp. were the taxa with the most abundant DEG functions at the species level. The functional categories were mainly carbohydrate transport and metabolism, transcription, lipid transport and metabolism, and coenzyme transport and metabolism. On the other hand, the MTP‐based functional groups consisted of 39 species from 15 families, with microbes from *Prevotellaceae* (38%), *Selenomonadaceae* (10%), *Lachnospiraceae* (10%), and *Succinibrionaceae* (8%) being the most abundant (Figure 3B and Table S2). *S. bovis* and *P. bryantii* were the taxa with the most abundant functions of DEPs at the species level.

Metabolite‐based functional groups (MTB‐based) were generated using metabolite origin analysis and origin‐based metabolic function analysis. Our rumen MTB data set was initially categorized into four groups, including 50 microbial metabolites, 4 host‐specific metabolites, 114 microbial‐host cometabolites, and 117 others (drug‐ and food‐related metabolites, as shown in Figure S2). After origin‐based functional analysis, 29 functions, including microbiota‐related and co‐metabolism pathways, were selected for downstream analysis of functional groups (Figure S2). Using the MetOrigin pipelines, biological relationships between the bacterial taxa and metabolites were visualized using a Sankey network. Our findings revealed that 10 KEGG pathways were biologically related to 25 microbial species from 12 families, with *S. ruminantium* exhibiting more differential functions than the other microbes (Figure 3C). The Sankey diagram mapping results for the rumen microbial metabolite‐based functional groups also demonstrated significant redundancy.

**Figure 4:**
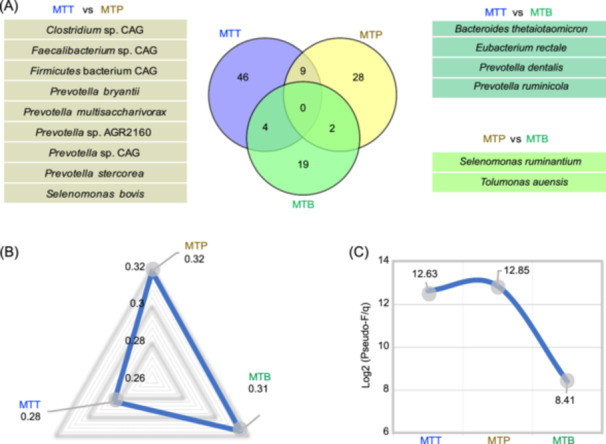
Comparison of rumen functional groups analyzed by different omics data. (A) Venn diagram showing overlaps of rumen microbial functional groups related to high feed efficiency based on MTT, MTP, and MTB. (B) Comparison of discriminatory power for feed efficiency of each functional group, as calculated by permutational multivariate analysis of variance (PERMANOVA) *R* ^2^ values. (C) Discriminatory power for feed efficiency of each functional groups as calculated by log_2_ (Pseudo‐*F*/*q*) values. Input values of omics data were determined by PERMANOVA of Bray‒Curtis‐based beta diversity. CAG, co‐abundance gene group.

We conducted a comparative analysis of three distinct levels of functional groups and assessed their compositional differences using a Venn diagram (Figure 4). We observed minimal overlap among the groups (Figure 4A), with the MTT and MTP groups sharing the highest number at 9 species, including *P. bryantii*, *S. bovis*, and three uncultured bacterial species from *Clostridium*, *Faecalibacterium*, and *Firmicutes*. To determine the significance of these differences in functional group composition among MTT, MTP, and MTB, we performed a permutational multivariate analysis of variance (PERMANOVA), revealing significant variations in composition between the two feed efficiency groups (*p* < 0.05). The *R* ^2^ values (0.28 for MTT, 0.32 for MTP, and 0.31 for MTB) indicated the proportion of variation in functional group composition explained by the grouping FCR factor (Figure 4B). We also calculated the effect size statistics, expressed as log_2_ (F/q), to quantify the proportion of total variation in the three functional groups that could be attributed to the FCR factor (Figure 4C). Our findings demonstrated that the feed efficiency phenotype had the most substantial impact on the total variation in MTP (12.85), followed by that in MTT (12.63), with the impact on that in MTB being the smallest (8.41).

### Rumen functional groups in high‐feed‐efficiency cows exerted important functions through key proteins

**Figure 5:**
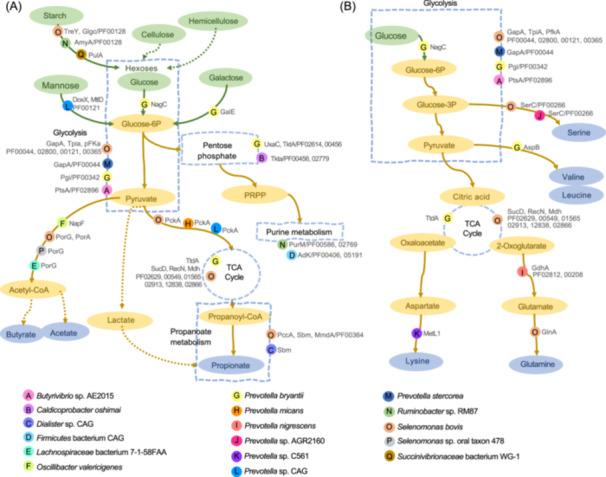
Pattern diagrams representing the role of key proteins involved in critical metabolic pathways in the rumen microbial functional groups based on the metaproteome. (A) Carbohydrates metabolism. (B) Nitrogen metabolism.

We found that the MTP‐based functional groups exhibited a distinct pattern of microbial participation in various metabolic pathways, with carbohydrate metabolism, amino acid metabolism, and bacterial chemotaxis being the most significant, as depicted in Figure 5. To gain a better understanding of the underlying mechanisms, we analyzed the key proteins and their domains involved in these pathways.

Carbohydrate metabolism, a complex process that involves various functional proteins and enzymes, includes glycolysis, pentose phosphate, purine metabolism, the tricarboxylic acid (TCA) cycle, and propanoate metabolism, as shown in Figure 5A. We identified four different microbes (*Selenomonas* sp., *Ruminobacter* sp., *Succinivibrionaceae* sp., and *Prevotella* sp.) that potentially function through alpha‐amylase, pullulanase, and transcription factors involved in starch and hexose degradation. Additionally, some of these microbes, such as *Selenomonas* sp., *Prevotella* sp., and *Butyrivibrio* sp., play a vital role in the catabolism of glucose to pyruvate through enzymes involved in the glycolytic pathway, such as glyceraldehyde‐3‐phosphate dehydrogenase. Furthermore, downstream pathways of carbohydrate metabolism were facilitated by functional groups that participate in acetyl‐CoA, propionate generation, and purine metabolism through several proteins, including membrane proteins of NapF, PorG, PorA, and RecN; enzymes of PckA, SucD, PccA, and Mdh; transport proteins of TtdA, Sbm, and MmdA; sugar metabolism proteins of UxaC and TktA; and metabolic regulation proteins of PurM and AdK. In addition, bacterial species such as *S. bovis* and *P. bryantii* (along with other *Prevotella* sp.) were found to participate in amino acid metabolism pathways through their enzymes, such as amino acid aminotransferase and decarboxylase, as illustrated in Figure 5B and Figure S3.

### Potentially causal relationships between *S. bovis* and functional proteins/metabolites contributed to high feed efficiency

**Figure 6:**
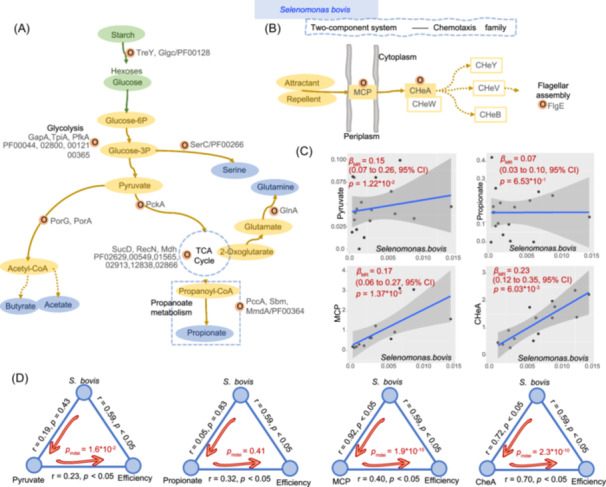
The proteins involved in the metabolic pathways in which *Selenomonas bovis* participates in the rumen function of high feed‐efficient dairy cows and their causal relationships with key functions. Proteins involved in key nutrient metabolism (A) and bacterial chemotaxis (B) functions played by *S. bovis* and their participating metabolic pathways. (C) Causal relationships between *S. bovis* and functional proteins/metabolites as assessed by Mendelian randomization (MR) analysis, with the MR effect size (*β*) and 95% confidence interval (CI) represented. (D) The direct mediation effects of *S. bovis* on feed efficiency through key functional proteins/metabolites, along with corresponding *p* values derived from mediation analysis (*p*_mdei_). The correlations between each factor are depicted by blue lines, showing Spearman coefficients and *p* values. We established a causal relationship between *S. bovis* and rumen carbohydrate metabolism, potentially mediated by bacterial chemotaxis and the two‐component regulatory system, ultimately impacting the feed utilization efficiency of dairy cows. CI, confidence interval; MCP, methyl‐accepting chemotaxis proteins.

Notably, *S. bovis* played a significant role in various pathways of carbohydrate metabolism (Figure 6A). Specifically, *S. bovis* potentially functioned in vital processes such as starch degradation (TreY), glycolysis (GapA), pentose phosphate (SucD, RecN, and Mdh), acetyl‐CoA formation (PorG, PorA), and propionate formation (PccA, Sbm, and MmdA), facilitated by its potential protein functions. Moreover, *S. bovis* species actively participated in bacterial chemotaxis pathways via crucial proteins such as the receptor that senses chemical gradients (methyl‐accepting chemotaxis protein, MCP), histidine kinase that autophosphorylates in response to environmental signals and transfers the phosphate to the response regulator protein (CHeA), and flagellar motor protein (FlgE), as shown in Figure 6B.

After associating *S. bovis* with MTP features that contribute to the high feed efficiency of animals, we utilized bidirectional Mendelian randomization (MR) and mediation analyses to determine whether *S. bovis* causally contributes to these features. At the protein level, we observed a positive correlation between the abundances of *S. bovis* and MCP (*r* = 0.92, *p* < 0.05) or CheA (*r* = 0.72, *p* < 0.05) using MR analysis (Figure 6C). These results were obtained at a significant effect size level (*β*) from the inverse variance weighted test (IVW) with *p* < 0.05. Additionally, the mediation analysis revealed that MCP and CheA could mediate the impact of *S. bovis* on the feed efficiency parameters (*p*_mdei_ < 0.05, Figure 6D). At the metabolic level, we also associated *S. bovis* with MTB features that contribute to the high feed efficiency of animals and conducted bidirectional MR and mediation analyses to assess whether *S. bovis* causally contributes to these features. Although we did not find any significant associations between *S. bovis* and MTB features, we identified a potentially positive causal relationship between *S. bovis* and pyruvate using MR analysis, with an effect size of IVW (*β*) = 0.15 (*p* < 0.05, Figure 6C). Our mediation analysis also indicated that pyruvate mediated the impact of *S. bovis* on feed efficiency (*p*_mdei_ < 0.05, Figure 6D).

Overall, by leveraging the rumen MTP, MTT, and MTB data, we overcame previous limitations to uncover the true functional distributions of rumen microorganisms and their impact on feed efficiency. Integrating host genetics data, we established potentially causal relationships among microbes, proteins/metabolites, and phenotypes. Specifically, we identified distinct functional patterns within rumen microorganism groups that influence host feed efficiency. Notably, we established a potentially causal relationship between *S. bovis* and rumen carbohydrate metabolism, potentially mediated by bacterial chemotaxis and the two‐component regulatory system, ultimately impacting the feed utilization efficiency of dairy cows. These findings elucidated the intricate interactions between the rumen microbiome and host phenotypes, contributing to our understanding of rumen microbial function and its implications for animal production.

## DISCUSSION

First, we used metaproteomics to study the real functional profiles of the rumen microbiota. Despite the initial proposal of metaproteomics early in 2004 [9] and ongoing advancements in detection [16, 17, 18, 19], database construction [20, 21, 22] and searching [23, 24], and analytical techniques [19, 25, 26, 27], research on the metaproteomics of rumen microbiota remains comparatively deficient, especially compared with other meta‐omics studies. A limited number of studies have focused on the rumen MTPs of adult sheep [28], preweaned lambs [29], cows [10, 11, 30], and moose [1]. While all metaproteomic studies of this nature to date have detected important microbial functions, they highlighted the active proteins and saccharide utilization loci by bacteria and fungi, with both deep functional resolution at the population level and broader system‐wide metabolic networks remaining undetected. Deusch et al. [11] provided deeper insight into the complicated microbial network of the rumen ecosystem and its response to different diets using three Jersey cows. In their work, a total of 8136 bacterial and 358 archaeal proteins were detected, and 166 carbohydrate active enzymes were revealed to present in varying abundances in animals fed corn silage, grass silage, or grass hay. A recent study showed that microbial protein production is tightly linked to the host feed efficiency phenotype, serving as a strong indicator of the host state phenotype, and it identified a total of 1629 core proteins [31]. In the current study, we employed high‐throughput proteomics to construct a greater number of rumen microbial proteomics datasets (a total of 19,167 proteins), surpassing the number of proteins identified in previous studies that reported the number of microbial proteins.

Although significant microbial functions have been detected in the rumen metaproteomic studies conducted so far, these studies have not had detailed functional resolution at the population level nor the broader system‐level metabolic networks that are necessary to ultimately link rumen microbiome function to host animal phenotypic traits [32]. Using holo‐omics, we investigated the functional guilds of rumen microbiota, those microbes that have a positive contribution to the metabolism of nutrients such as carbohydrates in high feed‐efficient dairy cows, at different levels and compared the similarities and differences among them, revealing significant FR. A previous study also investigated the redundancy of rumen microbiome functions [31]. They found that inefficient cows exhibited a greater degree of redundancy in their proteome than the efficient cows; conversely, efficient cows displayed higher levels of functional diversity and lower protein‐level competition. In contrast to their work, our research did not discuss high‐ and low‐feed efficiency cows separately. Instead, we focused specifically on high‐feed efficiency cows and found that, except for the protein level, the functions of microbial gene transcription and metabolite levels still exhibited significant redundancy. Within the human microbiome, despite the discernible functional variations and vast diversity of microbial genes that have been revealed via sophisticated computational metagenomic processing and meta‐analysis [33], the substantial functional overlap detected across individuals indicates the presence of considerable FR [34]. It has been reported that FR is important for stability and resilience [35, 36], but its origin and driving forces are poorly understood. A paradox exists due to different selection pressures from the host and microbial perspectives, which may drive FR in opposite directions. Host‐driven selection results in high FR, while microbial competition may result in low FR [37]. However, this paradox oversimplifies the spatial structure and environments of the human microbiome [38]. Low FR arises from divergent lineages with distinct gene suites, while high FR arises from a core set of genes needed for basic cellular functions. Although the concept of FR in rumen microbiota has been proposed by our current study and one previous study [31], the exploration of the complex rumen microbial communities and the degree of FR warrants investigation. Further research is essential to fully understand the intricate dynamics and implications of FR within rumen microbial communities.

Understanding the complex interactions and key functional groups within the rumen microbiome is essential for improving livestock production and reducing environmental impact [39, 40]. Researchers have identified numerous key functional groups in the rumen, including cellulolytic bacteria [1, 41, 42], methanogens [43, 44, 45], bacteria involved in the succinate pathway [46, 47], and lactate‐utilizing bacteria [48, 49], that play important roles in the breakdown of plant fibers, methane production, and the regulation of rumen pH. Additionally, researchers have identified complex interactions between different functional groups, including cross‐feeding and competition [1, 50], which are critical for maintaining the stability and efficiency of the rumen ecosystem. These findings have important implications for improving the efficiency of livestock production and reducing its environmental impact. In our study, we focused on identifying the core functional groups and understanding how these functional groups contribute to the potential and activity of the rumen ecosystem. The results presented in our study demonstrated the significant contribution of microbial functional groups in various metabolic pathways, particularly in carbohydrate metabolism, amino acid metabolism, and bacterial chemotaxis. Our analysis of key proteins and their domains involved in these functional groups revealed the involvement of several microbes, including *Selenomonas* sp., *Ruminobacter* sp., *Succinivibrionaceae* sp., and *Prevotella* sp. in the degradation of carbohydrates. Notably, *S. bovis* and *P. bryantii* emerged as crucial functional bacteria in the metabolism of carbohydrates (plant fibers), as well as amino acids. Potential reasons for the prominence of *S. bovis* and *P. bryantii* in carbohydrate and amino acid metabolism could include their enzymatic capabilities, genetic makeup, and ecological niche. *S. bovis* was noted for its involvement in a range of metabolic processes, encompassing starch breakdown, glycolysis, and the synthesis of pentose phosphate, acetyl‐CoA, and propionate [51]. Furthermore, our analysis unveiled the active engagement of *S. bovis* in bacterial chemotaxis pathways through key proteins like MCP, CHeA, and FlgE. These findings underscore the critical role of microbial functional groups in carbohydrate and amino acid metabolism and offer fresh insights into bacterial chemotaxis pathways. Additionally, our results also revealed that *P. bryantii* potentially functions in hexose degradation, pyruvate generation, and the TCA cycle. *P. bryantii* is a well‐studied bacterium that has been used as a probiotic in early‐lactation dairy cows and has shown beneficial effects on ruminal fermentation characteristics and milk production and composition [52]. These results indicate that *P. bryantii* not only functions in microbial ecology but also exerts regulatory potential on animal production performance. Despite the preliminary exploration of rumen FR in this study and previous research [31], attention should be given to the degree of FR within core functional groups. Future work should be conducted to investigate how this redundancy varies across different ruminant species, diets, and management practices, as well as the factors that shape the functional structure of these microbial communities. Our findings highlight the importance of further research into the FR of core microbial groups in the rumen to enhance our understanding and optimization of livestock production systems.

Researchers have investigated rumen microbiota‐host interactions and their impact on feed utilization efficiency [6, 7, 53]. Using metagenomic techniques, we previously studied the rumen microbiota under different interaction mechanisms and found that *S. bovis* is a key bacterium that positively interacts with succinate‐producing bacteria, leading to higher feed conversion efficiency [54]. Host genetic analysis also shows that *S. bovis* is a moderately heritable bacterium with important regulatory potential [55]. The role of *S. bovis* in improving feed conversion efficiency in ruminants can be attributed to several factors: positive interaction with succinate‐producing bacteria; genetic Influence; dominance in carbohydrate metabolism; specialized ecological niche. In our study, based on metatranscriptomics, we found that *S. bovis* dominates active carbohydrate metabolism functions. By analyzing the protein functional mechanisms and regulatory units of *S. bovis* at the protein level, we found that *S. bovis* significantly upregulates flagellum generation and bacterial chemotaxis, which significantly interact with carbohydrate utilization function. This indicates that *S. bovis* plays an important role in the rumen of high‐efficiency cattle through chemotaxis to carbon source substrates and by occupying the carbohydrate degradation niche.

Interestingly, we found that the “switch” (MCP) of the potential attractant receptor of the two‐component signal transduction system is significantly upregulated in the rumen of high‐efficiency dairy cows, with increased flagellar synthesis and chemotactic functions. These results suggest that special attractants can mediate the two‐component system of *S. bovis* via MCP receptors, thus regulating bacterial chemotaxis toward carbon source substrates and the occupation of carbohydrate ecological niches. We also identified that CheA and CheY from *S. bovis* were significantly upregulated in high feed efficiency animals, indicating the presence of a potential chemotaxis function of the classic chemotaxis gene cluster in the *S. bovis* two‐component system. Chemotaxis is a crucial adaptive behavior that allows microorganisms to thrive in complex environments and access various nutrient substrates. In high‐efficiency dairy cows, rumen microorganisms with chemotaxis can actively move toward specific nutrients, thereby enhancing nutrient utilization efficiency [56]. The MCP protein acts as a key sensor for bacterial chemotactic effectors, and is essential for detecting chemical signals, such as AI‐2, in the external environment. It can cross the cell membrane, and the periplasmic domain of MCP can sense external chemical signals, thereby activating downstream two‐component signal transduction systems, and changing the rotation direction of flagella to cause bacteria to exhibit chemotactic (or repellent) behavior [57]. The MCP is widely distributed in motile bacteria [58], with an average of 13.9 MCPs per bacterial genome, and the most extensively studied MCPs include Tar, Tsr, Tap, Trg, and Aer [59], while little is known about MCPs in rumen bacteria. During bacterial chemotaxis, MCP serves as the receptor, sensing signals in the two‐component system pathway [58]. Downstream response regulators such as CheA, CheW, CheY, and CheB, regulated by MCP, play crucial roles in signal transduction [57]. CheA phosphorylates the downstream response regulators CheY and CheB, enabling CheY‐P to affect flagellar rotation and achieve chemotaxis. This represents a more classical two‐component system‐mediated chemotaxis signaling pathway [60]. Although the regulation of bacterial chemotaxis signaling is not complex, the diversity of chemotaxis regulatory networks is considerable, and the functions of chemotaxis regulatory proteins have significant redundancy [61]. In addition, the existence of chemotaxis‐like gene clusters makes this regulatory network and its functions even more complex [62]. However, it is essential to investigate whether the classic chemotaxis gene cluster mediated by MCP exists in *S. bovis* and to understand the downstream signaling pathway. This can be achieved through advanced modern molecular biology and bioinformatics techniques, such as microbial pure culture methods, gene editing technology, and molecular docking at the strain level.

## CONCLUSION

In our study, we reported the protein‐level information in the rumen and integrated holo‐omics to explore functional groups and redundancy across RNA, protein, and metabolite levels. Additionally, we found a causal link between *Selenomonas bovis* and rumen carbohydrate metabolism, potentially mediated by bacterial chemotaxis and a two‐component regulatory system, impacting feed utilization efficiency of dairy cows. Our findings suggest a potentially nuanced understanding of the mapping of the rumen microbiota to its functional endpoints and its association with phenotypes. It's important to note that while our results provide valuable insights, caution is warranted when inferring causality from correlation data, and alternative explanations for observed associations should be considered to enhance scientific rigor. Our investigation into key nutrient functional groups in the rumen of high‐feed‐efficiency dairy cattle, as well as identifying key microbiota functional proteins, offers a foundation for transitioning from exploring correlations between omics findings to elucidating potentially causal relationships. This transition is crucial for facilitating precise regulation and manipulation of rumen microbiota. Moving forward, larger‐scale studies focusing on causal relationships, along with efforts to isolate and cultivate key functional bacteria, conduct mechanism studies at the strain level, and implement microbiota‐targeted modifications using synthetic biology, will further advance our understanding and lead to innovative applications building upon the insights gained from this study.

## METHODS

### Animals and samples

As reported by our previous study [54], a total of 57 lactating Holstein dairy cows were selected for FCR measurement, and 18 animals with 9 highest and 9 lowest FCR were selected for sampling of rumen contents. In the current study, we collected rumen metaproteomic data from these 18 animals with differential FCR.

### Isolation of ruminal proteins and peptides

The rumen content supernatant was collected by centrifugation at 300*g* and 1500*g*, respectively. The precipitates were added to four volumes of phenol extraction buffer (containing 10 mM dithiothreitol and 1% protease inhibitor) and then sonicated. An equal volume of Tris‐balanced phenol was added at 4°C, and the mixture was centrifuged at 5500*g* for 10 min. The supernatant was collected, five volumes of 0.1 ammonium acetate/methanol precipitation were performed, and then the samples were incubated overnight. The protein precipitates were washed with methanol and acetone. Finally, the precipitates were dissolved in 8 M urea, and the protein concentration was measured using a BCA protein assay kit (Thermo Fisher Scientific). Then, add standard samples (0, 5, 10, 15, 20 µL) to enzyme‐linked immunosorbent assay (ELISA) plate wells and top up each to 20 µL with sample diluent, triplicate each. Add 5 µL of protein sample to ELISA plate wells, top up to 20 µL with sample diluent, triplicate each. Then add 200 µL of BCA working solution to each well, incubate at 37°C for 30 min. Measure absorbance at 570 nm (other wavelengths between 562 nm and 595 nm are acceptable), and calculate protein concentration using standard curve and sample volume.

Equal amounts of proteins from each sample were taken and digested with trypsin. The volume of the lysate was adjusted to be the same, and 5 mM dithiothreitol (DTT) was added to reduce the disulfide bonds at 56°C for 30 min. Afterward, 11 mM iodoacetamide (IAA) was added and incubated in the dark at room temperature for 15 min. The urea was diluted with tetraethylammonium bromide to ensure a concentration below 2 M. Trypsin was added at a ratio of 1:50 (enzyme:protein, w/w), and the mixture was incubated overnight. Then, trypsin was added again at a ratio of 1:100 and digested for 4 h.

### LC‒MS/MS analysis and metaproteomic data collection

The peptides were dissolved in mobile phase A and separated using an EASY‐nLC 1200 ultrahigh‐performance liquid chromatography system (Thermo Fisher Scientific). Mobile phase A consisted of a water solution containing 0.1% formic acid and 2% acetonitrile, while mobile phase B was composed of a water solution containing 0.1% formic acid and 90% acetonitrile. The liquid phase gradient was programmed as follows: 0–90 min, with a linear increase from 5% to 25% mobile phase B; 90–112 min, with a linear increase from 25% to 35% mobile phase B; 112–116 min, with a rapid increase from 35% to 80% mobile phase B; and 116–120 min, maintaining 80% mobile phase B, with a constant flow rate of 500 nL/min.

Peptides were ionized by a nanospray ionization (NSI) source and then introduced into a Q Exactive™ Plus mass spectrometer (Thermo Fisher Scientific) for analysis. The ion source voltage was set at 2.1 kV. The parent ions and their fragments were detected and analyzed using a high‐resolution Orbitrap analyzer. The first mass spectrometry scan range was set to 350–1800 m/z with a scan resolution of 70,000, while the second mass spectrometry scan range started from 100 m/z with a scan resolution of 17,500. Data acquisition utilized a data‐dependent scanning program, where the top 20 peptide parent ions with the highest signal intensity after the first scan were selected for fragmentation using collision‐induced dissociation (CID) with 28% collision energy. Subsequently, these fragments underwent sequential secondary mass spectrometry analysis. To improve mass spectrometry efficiency, automatic gain control was set to 5E4, and the signal threshold was set to 4E4 ions/s. Additionally, the maximum injection time was set to 100 ms to optimize ion accumulation, and a dynamic exclusion time of 30 s was applied during tandem mass spectrometry scanning to prevent repeated scanning of precursor ions. Overall, these stringent experimental conditions were employed to ensure accurate peptide identification and quantification, as well as to maximize the sensitivity and reliability of the mass spectrometry analysis.

### Processing and bioinformatic analysis of metaproteomic data

The raw mass spectrometry data were imported into MaxQuant (v1.6.15.0) to search for the secondary mass spectrometry data. We used previously sequenced metagenomic data from 18 animals to prepare the proteomic search library [54]. To estimate the false discovery rate (FDR) due to random matches, a decoy database was incorporated, and a common contaminant database was included to eliminate contaminant proteins' influence on identification results. The following parameter settings were employed: enzyme cleavage specificity was set to trypsin/P; the maximum number of missed cleavage sites allowed was 2; the minimum length of peptide segments was set to 7 amino acid residues; and the maximum number of modifications per peptide segment was limited to 5. First‐level mass tolerances for the initial and main searches were set to 20 and 4.5 ppm, respectively, while the mass tolerance for second‐level fragment ions was set to 20 ppm. Carbamidomethyl (C) of cysteine was designated as a fixed modification, and variable modifications included methionine oxidation, protein N‐terminal acetylation, and deamidation (NQ). Both protein identification and peptide‐spectrum match (PSM) identification were subject to an FDR threshold of 1%. To ensure high‐quality analysis results, the accuracy of identification at the spectrum, peptide, and protein levels was set to an FDR of 1%, with identified proteins required to contain at least one unique peptide segment.

For GO enrichment analysis, proteins were classified by GO annotation into three categories: biological process, cellular compartment and molecular function. For each category, a two‐tailed Fisher's exact test was employed to test the enrichment of the DEPs against all identified proteins. GO terms with FDR‐corrected *p* values < 0.05 were considered significant. Both the KEGG and COG databases were used to identify enriched pathways by a two‐tailed Fisher's exact test to test the enrichment of the DEPs against all the identified proteins. A pathway with a corrected *p* value < 0.05 was considered significant. For each protein category, the InterPro database (a resource that provides functional analysis of protein sequences by classifying proteins into families and predicting the presence of domains and important sites) was utilized, and a two‐tailed Fisher's exact test was employed to test the enrichment of the DEPs against all identified proteins. Protein domains with a corrected *p* value < 0.05 were considered significant.

### Processing and bioinformatic analysis of metatranscriptomic and metabolomic data

Detailed metatranscriptomic data were reported in our previous study, with partial data reanalyzed in the current study [54]. Briefly, quality control, read filtering and trimming, sequence alignment and gene expression quantification were performed by using Trimmomatic (version 0.39), TopHat (version 2,1,1), Meta‐Velvet (version 1.2.01), and USEARCH (version 11), respectively. In this study, we used HUMAnN (version 2.0) to perform species‐level functional profiling based on the genome, gene, and pathway databases, including the UniRef, MetaCyc, MetaPhIAn2 and ChocoPhIAn pangenome databases. Trinotate (version 4.0.0) was employed to identify differentially expressed transcripts and to interactively explore the annotations and the expression data.

Rumen MTB from 18 dairy cattle with differential FCRs has been reported previously. Metabolite extraction method include: Transfer 100 μL of sample to a microcentrifuge tube, add 20 μL of urease (80 mg/mL), and incubate for 1 h. Add 360 μL of (methanol: ribose = 350:10) mixture, vortex, and ultrasound for 10 min in an ice water bath. Centrifuge the samples at 4°C and 12,000 revolutions per minute (RPM) for 15 min, then carefully transfer 180 μL of supernatant into a 1.5 mL microcentrifuge tube. Take 60 μL of each sample and combine them to create quality control (QC) samples. Dry the extract in a vacuum concentrator. After evaporation in a vacuum concentrator, add 80 μL of Methoxyamination hydrochloride (20 mg/mL in pyridine) and incubate at 80°C for 30 min, followed by derivatization with 100 μL of N, O‐bis (trimethylsilyl) trifluoroacetamide (BSTFA) regent (1% TMCS, v/v) at 70°C for 1.5 h. Gradually cool the samples to room temperature, then add 5 μL of FAMEs (in chloroform) to the QC sample.

Gas chromatography (Agilent Technologies) coupled to Pegasus HT time‐of‐flight/mass spectrometry (LECO Corporation), Chroma TOF software (version 4.3), and the LECO‐Fiehn Rtx5 database were used for metabolomic analysis and data processing. For detailed information: GC‐TOF‐MS analysis was conducted using an Agilent 7890 gas chromatograph coupled with a time‐of‐flight mass spectrometer. The system employed a DB‐5MS capillary column. A 1 μL aliquot of the sample was injected in splitless mode. Helium served as the carrier gas, with a front inlet purge flow of 3 mL min−1 and a gas flow rate of 1 mL min−1 through the column. The initial temperature was held at 50°C for 1 min, then ramped to 310°C at a rate of 10°C min−1, and maintained for 8 min at 310°C. The injection, transfer line, and ion source temperatures were set at 280, 280, and 250°C, respectively. The electron impact mode utilized an energy of −70 eV. Mass spectrometry data were acquired in full‐scan mode over the m/z range of 50–500 at a rate of 12.5 spectra per second following a solvent delay of 6.27 min.

In the current study, before downstream analyses, we discriminated the origins of microbial metabolites by using MetOrigin software (version 2022‐08) [63]. We used the SMOA mode from MetOrigin to quickly discriminate metabolites originating from the microbiome and performed origin‐based metabolic pathway enrichment analysis. We integrated statistical correlations and biological relationships.

### Generating functional groups

To generate the MTP‐based functional group information, enrichment‐based clustering was performed for hierarchical clustering based on DEP functional classification. We first collated all the COG categories obtained after enrichment along with their *p* values (higher in high FCR animals) and then filtered for those categories that were enriched in at least one of the clusters with a *p* value < 0.05. This filtered *p* value matrix was transformed by the function *x* = −log_10_ (*p* value). Finally, these *x* values were *z*‐transformed for each functional category. These *z* scores were then clustered by one‐way hierarchical clustering (Euclidean distance, average linkage clustering) in Genesis. Cluster membership was visualized by a heatmap using the “heatmap.2” function from the “gplots” R package. To generate the MTT‐based functional group, COG function categories were compared between two FCR groups using the Wilcoxon rank‐sum test, with those upregulated in high FCR animals selected (counts per million >5, FCR adjusted *p* < 0.05). To generate the MTB‐based functional group information, the MTB results generated from MetOrigin classification were used, with microbial‐related metabolites and those related to co‐metabolism by host and microbes used for downstream analysis. Origin‐based metabolic pathways were also analyzed by MetOrigin. Metabolite peaks that existed in over 50% of the samples, and with a similarity value of over 200 were selected for correlating with the FCR phenotype (Spearman correlation). Metabolites that were significantly higher in efficient animals (FCR adjusted *p* < 0.05) and were significantly correlated with FCR (*p* < 0.05) were considered functional metabolites from rumen microbes. Sankey diagrams were used to map the relationships among functional proteins/mRNAs/metabolites, microbial species, and taxonomic categories visualized by the “ggplot2” R package.

The comparisons among the three functional groups were performed by Venn diagram (Venny, version 2.1.0) and PERMANOVA. First, we calculated the Bray‒Curtis dissimilarity matrix based on the microbial community abundance data and performed PERMANOVA using the “adonis” function from the “vegan” package in R. Then, we set the dissimilarity matrix as the dependent variable and the grouping variable (FCR) as the independent variable and set the number of permutations (999) to generate a null distribution of the *F*‐statistic, *R*‐squared (variance explained) and *p* value. Finally, we performed post hoc tests (PERMDISP) to identify which groups differed significantly from each other.

### Genome‐wide association analysis for omics features

To investigate the causal effect using Two‐sample MR analysis behind omics‐based findings, a larger cohort containing 298 animals was used [55], with the 18 cows used in the current study contained in such a cohort. The SNP data, rumen microbiome data, together with MTB data were used for MR causal effect calculation. Herein, we performed microbial genome‐wide association studies (GWAS) using 179 animals SNP data and the abundance of microbes from the previously reported cohort [55]. Only microbes that were considered MTT functional groups were selected for the GWAS analysis. We also performed metabolite GWAS using another 119 animals that both contain SNP data and rumen metabolites data from the previously reported cohort [55]. Only microbial‐related metabolites that were significantly higher in high feed efficiency animals and correlated with FCR in the current study were chosen for the metabolite GWAS.

A total of 20,571 variants were used for GWAS. PLINK software (version 1.9) was used in this study for GWAS analysis. Data preparation was conducted as follows: The omics data were converted into PLINK format using SAMtools (version 1.17). QC of the data was carried out by removing variants with low minor allele frequency, low genotyping rate, high missingness, or significant deviation from Hardy‒Weinberg equilibrium. QC of the samples entailed removing those with high levels of missingness, population stratification, or relatedness, aimed to eliminate any potential biases or confounding factors that could affect the reliability of the results. The phenotype and genotype data, after quality control, were analyzed using the rrBLUP (version 4.6.1) package in R with the following model: y = 1λ + *Z a* + *J g* + *e*, where *y* represents the adjusted values of microbial taxa; *a* and *e* are the random additive genetic effect and the random residual effect, respectively, with assumptions of distribution, variance, and covariance structure as described above; *Z* is the design matrix; *g* is a fixed effect modeling the additive SNP effect; and *J* is the design matrix. Genotypes were coded as −1, 0, and 1 for AA, AB, and BB, respectively. The Benjamini‐Hochberg method was used to adjust the *p*‐values (*p‐adj*) calculated for each trait.

### Potentially causal effect between functional microbes and proteins/metabolites

To estimate whether microbes can causally contribute to proteins as well as metabolites, we performed MR analyses, focusing on the bacterium *S. bovis*. Based on the microbial GWAS summary statistics data for the microbiota, 28 independent genetic variants with *p‐adj* < 0.001 were selected as instrumental variables and used for two‐sample MR analysis. In addition, the metabolite GWAS summary statistics data for the metabolites (independent genetic variants with *p* < 0.001) that were significantly associated with FCR were selected for the two‐sample MR analysis. The MR analysis was performed using TwoSampleMR (version 0.5.6) in the R project, with five methods (IVW [64], MR‒Egger regression [65], weighted median [66], and mode‐based estimate including simple mode and weighted mode [67]). IVW combines the effect estimates of each genetic variant, weighted by the inverse of their variance, providing a meta‐analytic estimate of the causal effect [64].

MR‐Egger regression assesses the presence of directional pleiotropy and provides a causal estimate adjusted for this bias [65]. Weighted median calculates a median‐based estimate that remains consistent even if up to 50% of the genetic variants are invalid instruments [66]. Both simple mode, and weighted mode of mode‐based methods were applied, which cluster genetic variants into groups based on similar causal estimates and derive an overall causal effect from the largest cluster [67]. In addition to the MR analysis, we conducted a causal mediation analysis to further explore the potential pathways through which *S. bovis* might influence protein and metabolite levels. This analysis was performed using the mediation package (version 4.5.0) in R. The mediation analysis quantifies the extent to which the effect of an independent variable on a dependent variable is mediated by an intermediate variable [68]. In the current study, the process involved: fitting models to estimate the direct and indirect effects of *S. bovis* on the proteins and metabolites; and then testing the significance of these mediation effects to understand the contribution of intermediate processes.

## AUTHOR CONTRIBUTIONS

**Ming–Yuan Xue**: Methodology, investigation, data curation, writing—original draft preparation; **Yun–Yi Xie**: Investigation, data curation; **Xin–Wei Zang**: Investigation, data curation; **Yi–Fan Zhong**: Data curation and visualization; **Xiao–Jiao Ma**: Data curation; **Hui–Zeng Sun**: Supervision, project administration, writing—reviewing and editing; **Jian–Xin Liu**: Funding acquisition, supervision, writing—reviewing and editing.

## CONFLICT OF INTEREST STATEMENT

The authors declare no conflict of interest.

## ETHICS STATEMENT

Animal care and experimental procedures (No. ZJU20170422) were approved by the Animal Care Committee of Zhejiang University (Hangzhou, China) and followed the university's guidelines for animal research.

## Supporting information


**Figure S1:** Cluster results to identify functional correlations among differentially expressed proteins in comparison groups after performing GO classification and enrichment of differentially expressed proteins in various comparison groups.
**Figure S2:** Metabolomics‐based rumen microbial functional groups related to high feed efficiency in dairy cows.
**Figure S3:** The proteins involved in the metabolic pathways in which Prevotella bryantii participates in the rumen function of high feed efficiency dairy cows.

**Table S1:** Phenotypic information and rumen fermentation parameters of dairy cows.
**Table S2:** Functional groups of ruminal metatranscriptomics (MTT), metaproteomics (MTP), and metabolomics (MTB).

## Data Availability

Sequencing data were deposited into the NCBI Sequence Read Archive (SRA) under the accession number of PRJNA597489 (https://www.ncbi.nlm.nih.gov/bioproject/PRJNA597489/). The genetic data are available at the Genome Variation Map (GVM) in China National Center for Bioinformation under the accession number of GVM000134 (https://ngdc.cncb.ac.cn/gvm/getProjectDetail?project=GVM000134) and GVM000274 (https://ngdc.cncb.ac.cn/gvm/getProjectDetail?project=GVM000274). Data and scripts used were saved in GitHub (https://github.com/Anim-Microb/iMeta-XUE-2024). Supplementary materials (figures, tables, graphical abstract, slides, videos, Chinese translated version, and update materials) can be found in the online DOI or iMeta Science (http://www.imeta.science/).
