# Supplementary material for: Deciphering functional groups of rumen microbiome and their underlying potentially causal relationships in shaping host traits
Source: Imeta. 2024 Jul 15;3(4):e225. doi: 10.1002/imt2.225 (PMC11316931; doi:10.1002/imt2.225)
Supplement: Supplementary file 1 — Figure S1: Cluster results to identify functional correlations among differentially expressed proteins in comparison groups after performing GO classification and enrichment of differentially expressed proteins in various comparison groups. Figure S2: Metabolomics‐based rumen microbial functional groups related to high feed efficiency in dairy cows. Figure S3: The proteins involved in the metabolic pathways in which Prevotella bryantii participates in the rumen function of high feed efficiency dairy cows. [file IMT2-3-e225-s002.docx]

**Supporting information to**

**Deciphering functional groups of rumen microbiome and their underlying potentially causal relationships in shaping host traits**

**Running title**: Functional groups and causal relationships of rumen microbiome in shaping host traits

Ming-Yuan Xue^1,2^, Yun-Yi Xie^1^, Xin-Wei Zang^1^, Yi-Fan Zhong^1^, Xiao-Jiao Ma^1^, Hui-Zeng Sun^1,3^, Jian-Xin Liu^1,3^

^1^Institute of Dairy Science, College of Animal Sciences, Zhejiang University, Hangzhou 310058, China

^2^Xianghu Laboratory, Hangzhou 311231, China

^3^Ministry of Education Key laboratory of Molecular Animal Nutrition, Zhejiang University, Hangzhou 310058, China

Correspondence: [huizeng@zju.edu.cn](mailto:huizeng@zju.edu.cn) (Hui-Zeng Sun), [liujx@zju.edu.cn](mailto:liujx@zju.edu.cn) (Jian-Xin Liu)


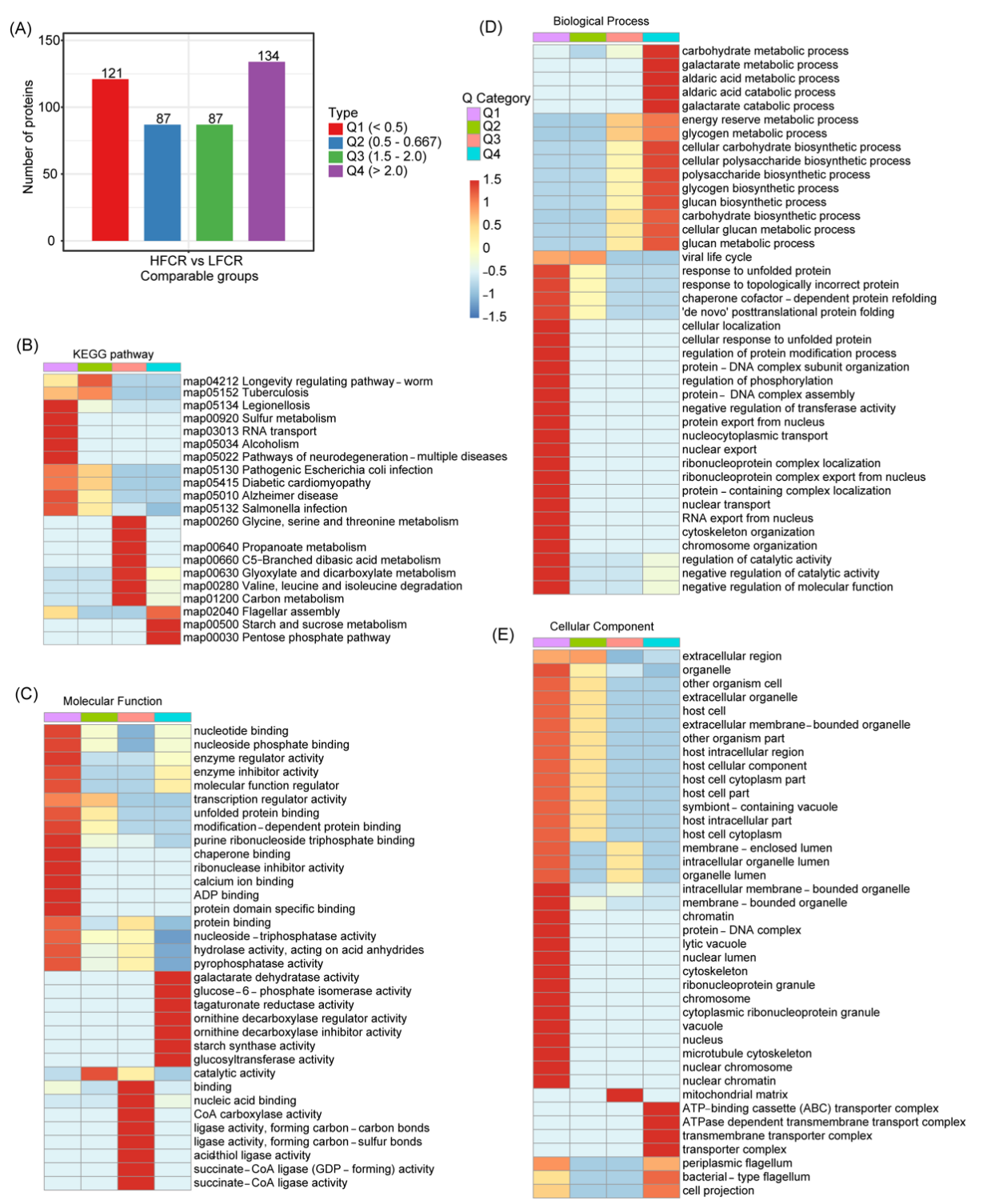


**Figure S1** Cluster results to identify functional correlations among differentially expressed proteins in comparison groups after performing GO classification and enrichment of differentially expressed proteins in various comparison groups. (A) Comparison of clusters in the two groups. (B) Heatmap showing classification of pathways. (C) Heatmap showing classification of molecular functions. (D) Heatmap showing classification of biological process functions. (E) Heatmap showing classification of cellular component functions.

**
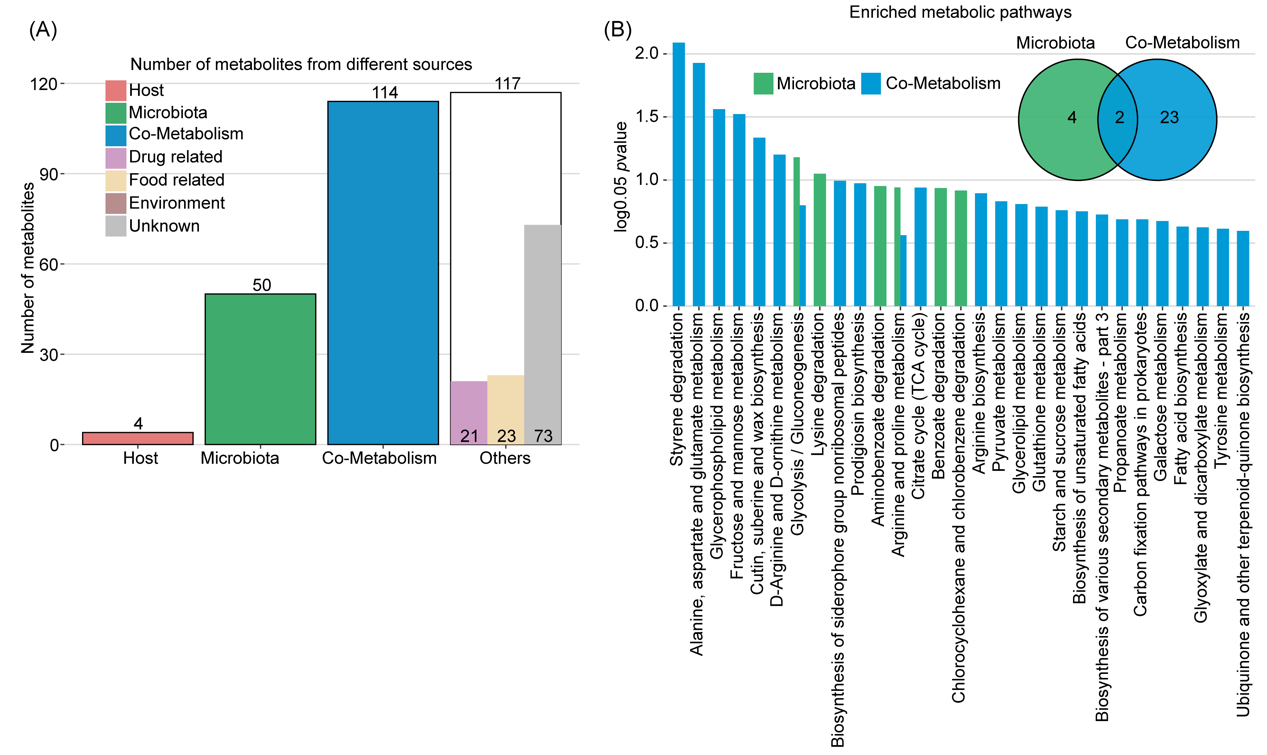
**

**Figure S2** Metabolomics-based rumen microbial functional groups related to high feed efficiency in dairy cows. (A) Discriminating the origins of rumen microbial metabolites from the bulk metabolome using the MetOrigin workflow. Bar plot representing the number of metabolites from the host, microbiota, and metabolites involved in the cometabolism of both the host and microbiota, together with drug-related, food-related and environment-related metabolites. (B) Metabolic pathway enrichment analysis according to different origins, including both microbial and cometabolism metabolites.


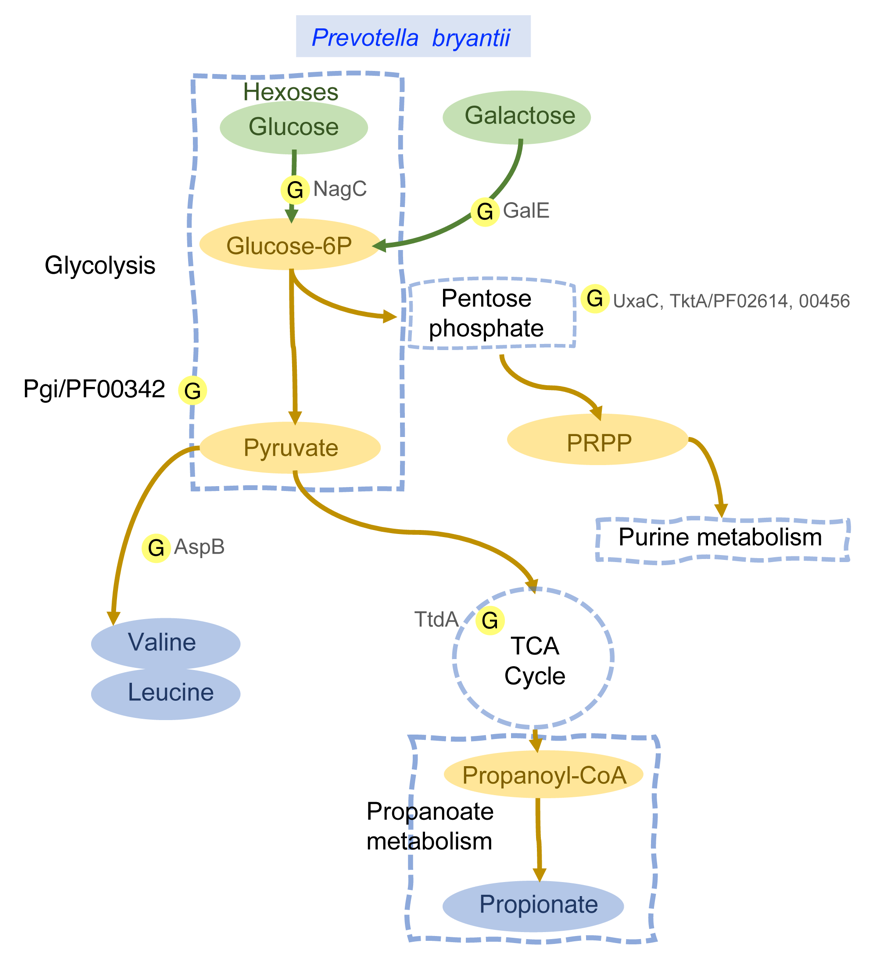


**Figure S3** The proteins involved in the metabolic pathways in which *Prevotella bryantii* participates in the rumen function of high feed efficiency dairy cows.
